# Leber Congenital Amaurosis Associated with Mutations in *CEP290*, Clinical Phenotype, and Natural History in Preparation for Trials of Novel Therapies

**DOI:** 10.1016/j.ophtha.2017.12.013

**Published:** 2018-06

**Authors:** Leo Sheck, Wayne I.L. Davies, Phillip Moradi, Anthony G. Robson, Neruban Kumaran, Alki C. Liasis, Andrew R. Webster, Anthony T. Moore, Michel Michaelides

**Affiliations:** 1 Moorfields Eye Hospital, London, United Kingdom; 2 UWA Oceans Institute, University of Western Australia, Crawley, Australia; 3 School of Biological Sciences, University of Western Australia, Crawley, Australia; 4 Lions Eye Institute, University of Western Australia, Crawley, Australia; 5 University College London Institute of Ophthalmology, University College London, United Kingdom; 6 Great Ormond Street Hospital, London, United Kingdom; 7 Ophthalmology Department, School of Medicine, University of California San Francisco, San Francisco, California

**Keywords:** CFP, color fundus photography, ERG, electroretinogram, EOSRD, early-onset severe retinal dystrophy, FAF, fundus autofluorescence, LCA, Leber congenital amaurosis, PERG, pattern electroretinogram, SD, standard deviation, VA, visual acuity

## Abstract

**Purpose:**

To investigate and describe in detail the demographics, functional and anatomic characteristics, and clinical course of Leber congenital amaurosis (LCA) associated with mutations in the *CEP290* gene (LCA-*CEP290*) in a large cohort of adults and children.

**Design:**

Retrospective case series.

**Participants:**

Patients with mutations in *CEP290* identified at a single UK referral center.

**Methods:**

Review of case notes and results of retinal imaging (color fundus photography, fundus autofluorescence [FAF] imaging, OCT), electrophysiologic assessment, and molecular genetic testing.

**Main Outcome Measures:**

Molecular genetic testing, clinical findings including visual acuity and retinal imaging, and electrophysiologic assessment.

**Results:**

Forty patients with LCA-*CEP290* were identified. The deep intronic mutation c.2991+1655 A>G was the most common disease-causing variant (23/40 patients) identified in the compound heterozygous state in 20 patients (50%) and homozygous in 2 patients (5%). Visual acuity (VA) varied from 6/9 to no perception of light, and only 2 of 12 patients with longitudinal VA data showed deterioration in VA in their better-seeing eye over time. A normal fundus was found at diagnosis in younger patients (mean age, 1.9 years), with older patients showing white flecks (mean age, 5.9 years) or pigmentary retinopathy (mean age, 21.7 years). Eleven of 12 patients (92%) with OCT imaging had preservation of foveal architecture. Ten of 12 patients (83%) with FAF imaging had a perifoveal hyperautofluorescent ring. Having 2 nonsense *CEP290* mutations was associated with worse final VA and the presence of nonocular features.

**Conclusions:**

Detailed analysis of the clinical phenotype of LCA-*CEP290* in a large cohort confirms that there is a window of opportunity in childhood for therapeutic intervention based on relative structural preservation in the central cone-rich retina in a significant proportion of patients, with the majority harboring the deep intronic variant potentially tractable to several planned gene editing approaches.

Leber congenital amaurosis (LCA) was first described by Theodore Leber in 1869 and refers to a heterogeneous group of retinal disorders with early-onset vision loss, nystagmus, and an extinguished electroretinogram (ERG).^1^ Leber later described a separate group of milder disease phenotypes, with some preservation of the ERG responses (now referred to as “early-onset severe retinal dystrophy” [EOSRD] or “severe early childhood onset retinal dystrophy”).^2^, ^3^ There is considerable clinical and genetic overlap between LCA and EOSRD/severe early childhood onset retinal dystrophy. Leber congenital amaurosis and EOSRD account for a significant proportion of blindness in children worldwide,^4^, ^5^, ^6^ with an annual estimated incidence of 1 in 30 000 newborns.^7^ In the United Kingdom, 14% of children with newly diagnosed blindness have LCA/EOSRD.^8^

Twenty-five causative genes have been identified to date, accounting for 70% to 80% of all LCA/EOSRD cases. *CEP290* is one of the most common causes, accounting for 15% to 20% of all known cases.^9^ The intronic variant c.2991+1655A>G is the most common pathogenic mutation, especially in Europe and the United States,^7^ identified in 77% of all patients in 1 cohort with *CEP290*-related disease.^10^

*CEP290* encodes a protein that localizes to the transition zone of the connecting cilium, including the cilia of photoreceptors^11^; Leber congenital amaurosis-*CEP290* is one of an increasing number of retinal dystrophies that can be classified as a ciliopathy.^9^, ^12^ In addition to isolated LCA/EOSRD, *CEP290* mutations also have been identified in Bardet–Biedl syndrome, Senior–Loken syndrome, Joubert syndrome, and Meckel–Gruber syndrome.^7^ No definitive genotype-phenotype correlation has been established for isolated ocular versus syndromic *CEP290*-associated disease.^3^, ^13^

Since the development of gene-based therapy for *RPE65*-associated LCA/EOSRD, there has been considerable interest in novel treatments for other molecular forms of LCA/EOSRD.^3^, ^14^ Lentiviral vector gene replacement, antisense oligonucleotide, and *CRISPR*/Cas9-based techniques are all under active investigation as viable interventions in *CEP290* LCA/EOSRD.^15^, ^16^, ^17^, ^18^, ^19^

The current study provides a detailed characterization of the clinical phenotype and natural history in a large number of patients with *CEP290* LCA/EOSRD seen at a single institution, which will help to provide improved genetic counseling and advice on prognosis, and to assist in the preparation and design of anticipated clinical trials of novel therapies.

## Methods

### Patient Identification and Assessment

Patients harboring likely disease-causing variants in *CEP290* were identified from the Moorfields Eye Hospital Inherited Eye Disease database. Patients were included in this database after obtaining informed consent. This retrospective study adhered to the tenets of the Declaration of Helsinki and was approved by the Moorfields Eye Hospital ethics committee.

Clinical notes, imaging, and electrophysiologic testing were reviewed. Clinical data extracted included visual acuity (VA), subjective and objective refraction, slit-lamp biomicroscopy, and fundoscopy findings. OCT, fundus autofluorescence (FAF) imaging, and color fundus photography were reviewed when available. Fundus photographs were obtained with a TRC-50LA Retinal fundus camera (Topcon, Tokyo, Japan) or Optos wide-field camera (Optos Panoramic 200; Optos PLC., Scotland, UK). Fundus autofluorescence images were obtained with Spectralis HRA OCT (Heidelberg Engineering, Heidelberg, Germany) or Optos wide-field camera. Retinal lamination and central retinal thickness were evaluated using the Spectralis HRA OCT (Heidelberg Engineering). Full-field and pattern electroretinogram (PERG) incorporated the International Society for Clinical Electrophysiology of Vision standards,^20^, ^21^ except in young patients who underwent ERG testing with skin electrodes without mydriasis, using a previously reported protocol.^22^

### Molecular Diagnosis

The majority of patients were screened using a diagnostic targeted next-generation sequencing panel for retinal dystrophy. Others were ascertained via research-based whole exome sequencing or the Asper microarray chip (Asper, Tartu, Estonia), or in targeted Sanger sequencing of *CEP290*. All patients with 1 allele identified from the Asper chip were subjected to Sanger sequencing to identify the second allele.

## Results

### Molecular Genetics

**Figure 1:**
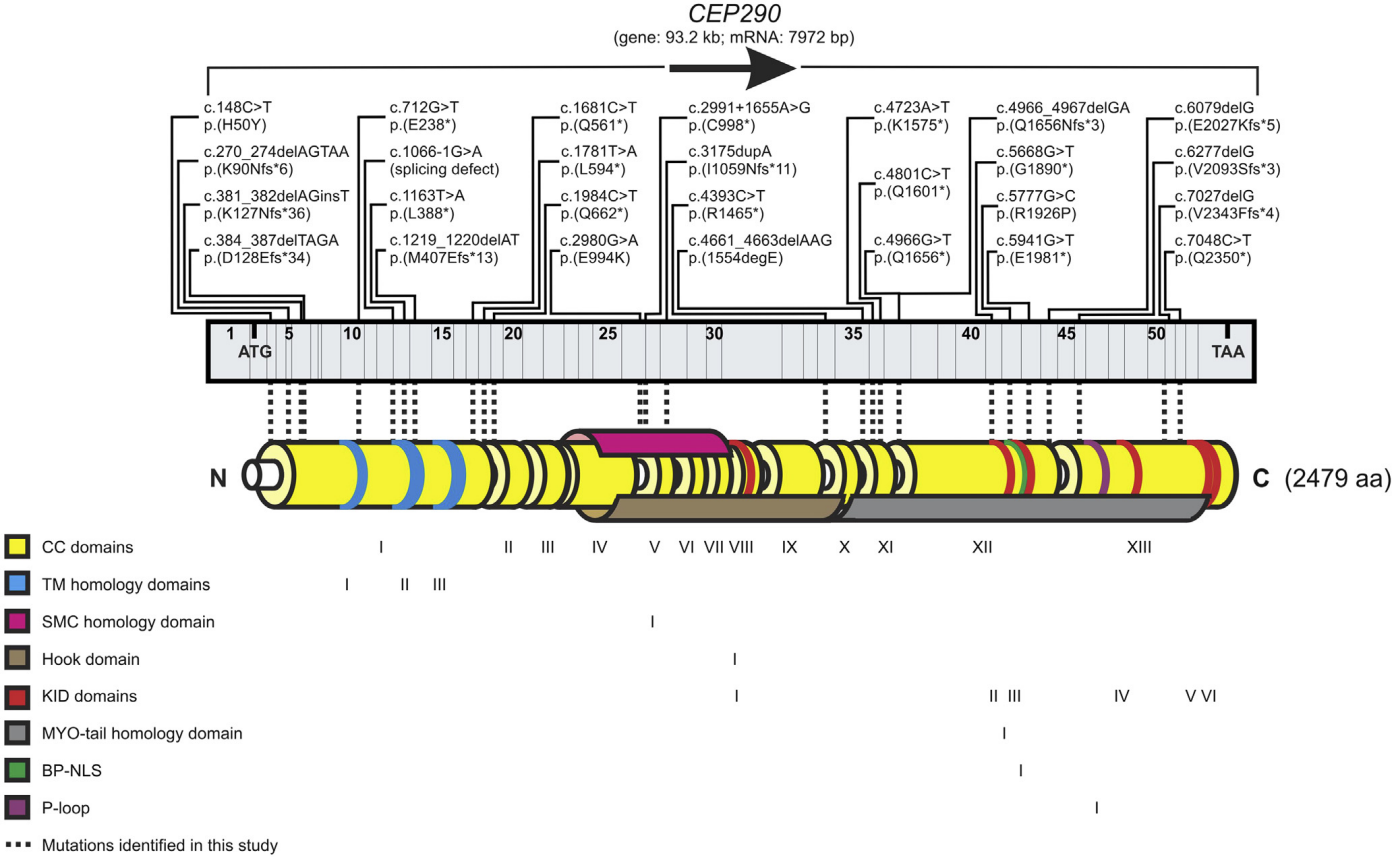
Schematic diagram showing the *CEP290* gene and the location of the mutations identified in our cohort. LCA = Leber congenital amaurosis.

Table 1 and Figure 1 detail the molecular findings in our cohort of 40 LCA-*CEP290* patients, including 3 families with multiple affected members (patients 5a to 5e, 22a and 22b, and 31a to 31c), with the remaining patients being simplex cases. Grantham score is provided when appropriate.^23^ The majority of patients had 2 *CEP290* variants identified (n = 36, 90%), with the rest having 1 mutation identified to date (n = 4, 10%). The deep intronic mutation c.2991+1655A>G was the most common mutation (23/40 patients; 57.5%), identified in the compound heterozygous state in 20 patients (50%) and homozygous in 2 patients (5%). The effects of the *CEP290* mutations identified in our cohort are summarized in Table 2. The majority (75%) of variants encoded for premature stop codons, which would lead to protein truncation and dysfunction due to the loss of critical CEP290 functional domains.^11^ More important, premature stop codons result in nonsense-mediated decay, which would remove these aberrant transcripts, thus significantly decreasing the steady-state of CEP290 mRNA levels.^24^ Conversely, only 12.5% of variants result in missense amino acid substitutions, namely, p.(His50Tyr) (a positive to a neutral charge), p.(Glu994Lys) (a negative to a positive charge), and p.(Arg1926Pro) (a positive to a neutral charge). Given that all 3 substitutions constitute changes in the net charge of the amino acid side changes, they are likely to induce significant effects on CEP290 function.

**Table 1:** Molecular Findings in the Study Leber Congenital Amaurosis *CEP290* Cohort

Patient No.	GC No.	Mutation 1	Effect	Grantham Score†	Mutation 2	Effect	Grantham score†
1	17585	c.2991+1655A>G	p.(Cys998∗)	n/a	c.2980G>A	p.(Glu994Lys)	56 (probably tolerated, but this missense mutation results in a change of charge from negative to positive that may render the CEP290 protein functionless)
2	18665	c.2991+1655A>G	p.(Cys998∗)	n/a	unknown	unknown	n/a
3	17243	c.2991+1655A>G	p.(Cys998∗)	n/a	c.1163T>A	p.(Leu388∗)	n/a
4	14293	c.2991+1655A>G	p.(Cys998∗)	n/a	c.2991+1655A>G	p.(Cys998∗)	n/a
5a	1874	c.4393C>T	p.(Arg1465∗)	n/a	c.148C>T	p.(His50Tyr)	83 (possibly not tolerated)
5b	1874	c.4393C>T	p.(Arg1465∗)	n/a	c.148C>T	p.(His50Tyr)	83 (possibly not tolerated)
5c	1874	c.4393C>T	p.(Arg1465∗)	n/a	c.148C>T	p.(His50Tyr)	83 (possibly not tolerated)
5d	1874	c.4393C>T	p.(Arg1465∗)	n/a	c.148C>T	p.(His50Tyr)	83 (possibly not tolerated)
5e	1874	c.4393C>T	p.(Arg1465∗)	n/a	c.148C>T	p.(His50Tyr)	83 (possibly not tolerated)
6	16827	c.2991+1655A>G	p.(Cys998∗)	n/a	c.1984C>T	p.(Gln662∗)	n/a
7	19073	c.4723A>T	p.(Lys1575∗)	n/a	c.712G>T	p.(Glu238∗)	n/a
8	19328	c.2991+1655A>G	p.(Cys998∗)	n/a	unknown	unknown	n/a
9	17668	c.2991+1655A>G	p.(Cys998∗)	n/a	c.6277delG	p.(Val2093fs)	n/a
10	18259	c.4723A>T	p.(Lys1575∗)	n/a	c.4966G>T	p.(Glu1656∗)	n/a
11	18410	c.2991+1655A>G	p.(Cys998∗)	n/a	c.3175dupA	p.(Ile1059Asnfs∗11)	149‡ (probably not tolerated)
12	16596	c.2991+1655A>G	p.(Cys998∗)	n/a	c.2991+1655A>G	p.(Cys998∗)	n/a
13	17341	c.4723A>T	p.(Lys1575∗)	n/a	c.6079delG	p.(Glu2027Lysfs∗5)	56‡ (probably tolerated)
14	19709	c.2991+1655A>G	p.(Cys998∗)	n/a	c.1781T>A	p.(Leu594∗)	n/a
15	17947	c.2991+1655A>G	p.(Cys998∗)	n/a	c.384_387delTAGA	p.(Asp128Glufs∗34)	45‡ (probably tolerated)
16	19085	c.2991+1655A>G	p.(Cys998∗)	n/a	unknown	unknown	n/a
17	18805	c.2991+1655A>G	p.(Cys998∗)	n/a	c.1066-1G>A	splice	n/a
18	19043	c.2991+1655A>G	p.(Cys998∗)	n/a	c.4966G>T	p.(Glu1656∗)	n/a
19	18444	c.2991+1655A>G	p.(Cys998∗)	n/a	c.4723A>T	p.(Lys1575∗)	n/a
20	18269	c.5668G>T	p.(Gly1890∗)	n/a	c.5668G>T	p.(Gly1890∗)	n/a
21	23097	c.1681C>T	p.(Gln561∗)	n/a	c.7027delG	p.(Val2343Phefs∗4)	50‡ (probably tolerated)
22a	15931	c.5777G>C	p.(Arg1926Pro)	103 (probably not tolerated)	c.4966_4967delGA	p.(Glu1656Asnfs∗3)	42‡ (probably tolerated)
22b	15931	c.5777G>C	p.(Arg1926Pro)	103 (probably not tolerated)	c.4966_4967delGA	p.(Glu1656Asnfs∗3)	42‡ (probably tolerated)
23	17147	c.2991+1655A>G	p.(Cys998∗)	n/a	c.381_382delAGinsT	p.(Lys127Asnfs36∗)	94‡ (possibly not tolerated)
24	16858	c.2991+1655A>G	p.(Cys998∗)	n/a	c.1219_1220delAT	p.(Met407Glufs∗13)	126‡ (probably not tolerated)
25	23818	c.2991+1655A>G	p.(Cys998∗)	n/a	c.7048C>T	p.(Gln2350∗)	n/a
26	18721	c.3175dupA	p.(Ile1059Asnfs∗11)	149‡ (probably not tolerated)	unknown	unknown	n/a
27	13786	c.2991+1655A>G	p.(Cys998∗)	n/a	c.4966G>T	p.(Glu1656∗)	n/a
28	18481	c.2991+1655 A>G	p.(Cys998∗)	n/a	c.5941G>T	p.(Glu1981∗)	n/a
29	19641	c.2991+1655A>G	p.(Cys998∗)	n/a	c.4801C>T	p.(Gln1601∗)	n/a
30	16829	c.148C>T	p.His50Tyr	83 (possibly not tolerated)	c.148C>T	p.(His50Tyr)	83 (possibly not tolerated)
31a	24072	c.4661_4663delAAG	p.(1554delGlu)	n/a	c.4661_4663delAAG	p.(1554delGlu)	n/a
31b	24072	c.4661_4663delAAG	p.(1554delGlu)	n/a	c.4661_4663delAAG	p.(1554delGlu)	n/a
31c	24072	c.4661_4663delAAG	p.(1554delGlu)	n/a	c.4661_4663delAAG	p.(1554delGlu)	n/a
32	24225	c.2991+1655A>G	p.(Cys998∗)	n/a	c.270_274delAGTAA	p.(Lys90Asnfs∗6)	94‡ (possibly not tolerated)
33	25255	c.2991+1655A>G	p.(Cys998∗)	n/a	c.3175dupA	p.(Ile1059Asnfs∗11)	149‡ (probably not tolerated)

c. = coding region; del = deletion; dup = duplication; fs = frameshift; fs*digit = frameshift that results in a translation termination codon occurring downstream at the designated number of amino acids; n/a = not applicable; p. = protein.

∗ Translation termination codon.

† Grantham scoring, where <60 = probably tolerated; 61–99 = possibly not tolerated; >100 = probably not tolerated.

‡ Despite the fact that a missense mutation may be assigned a Grantham score, these mutations result in frameshifts that truncated the CEP290 protein, thus rendering the gene product functionless.

**Table 2:** Predicted Effect of *CEP290* Mutations

Predicted Effect	No. (Total = 80, 2 Alleles per Patient)	Frequency
Termination	60	75%
Substitution (missense)	10	12.5%
In-frame amino acid deletion	6	7.5%
Second mutation unknown	4	5%

### Clinical Findings

**Figure 2:**
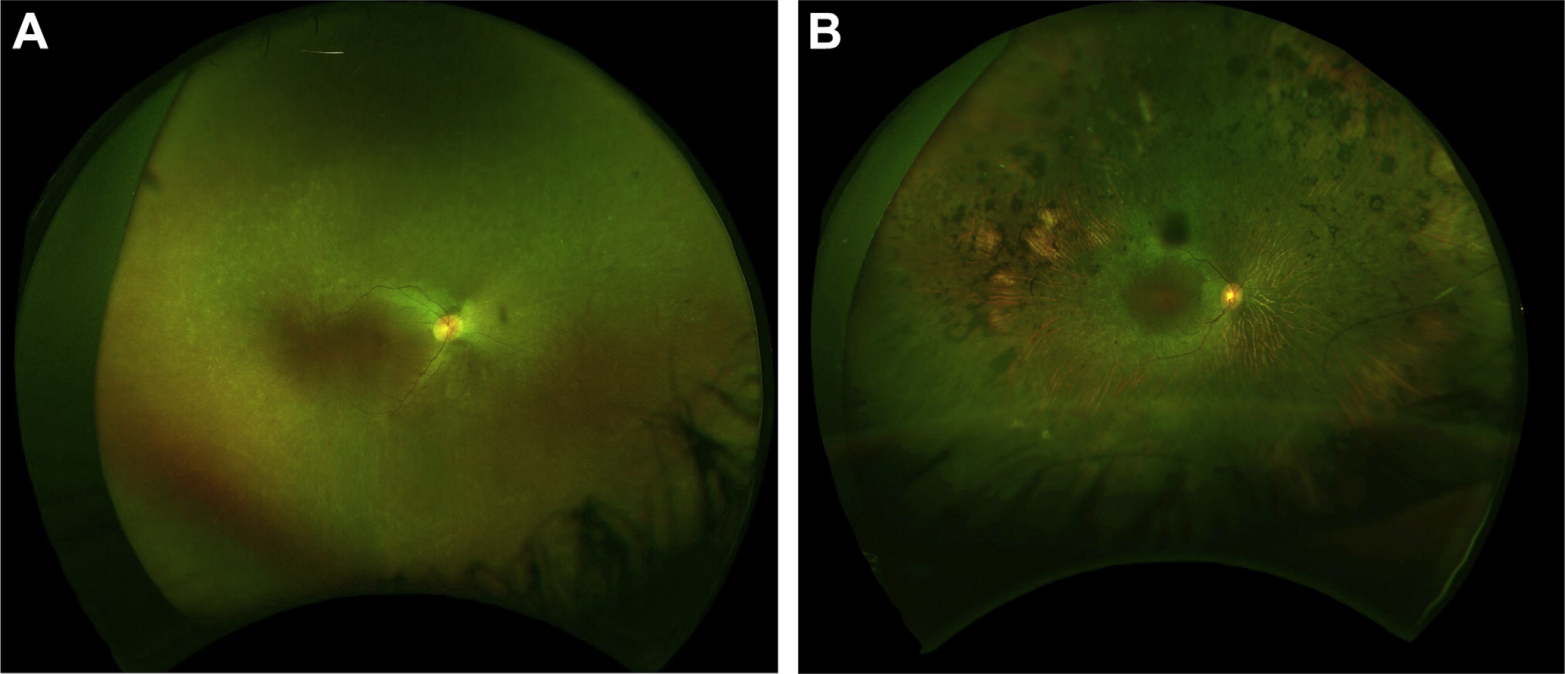
Optos (Optos Panoramic 200; Optos PLC., Scotland, UK) color fundal image of the right eye of patient 31a **(A)** showing the typical white flecks found in *CEP290* Leber congenital amaurosis (LCA). Very mild pigmentary changes were also observed. This is compared with the Optos color fundal image of the right eye of patient 5e **(B)** showing severe peripheral retinal atrophy and pigment formation.

**Figure 3:**
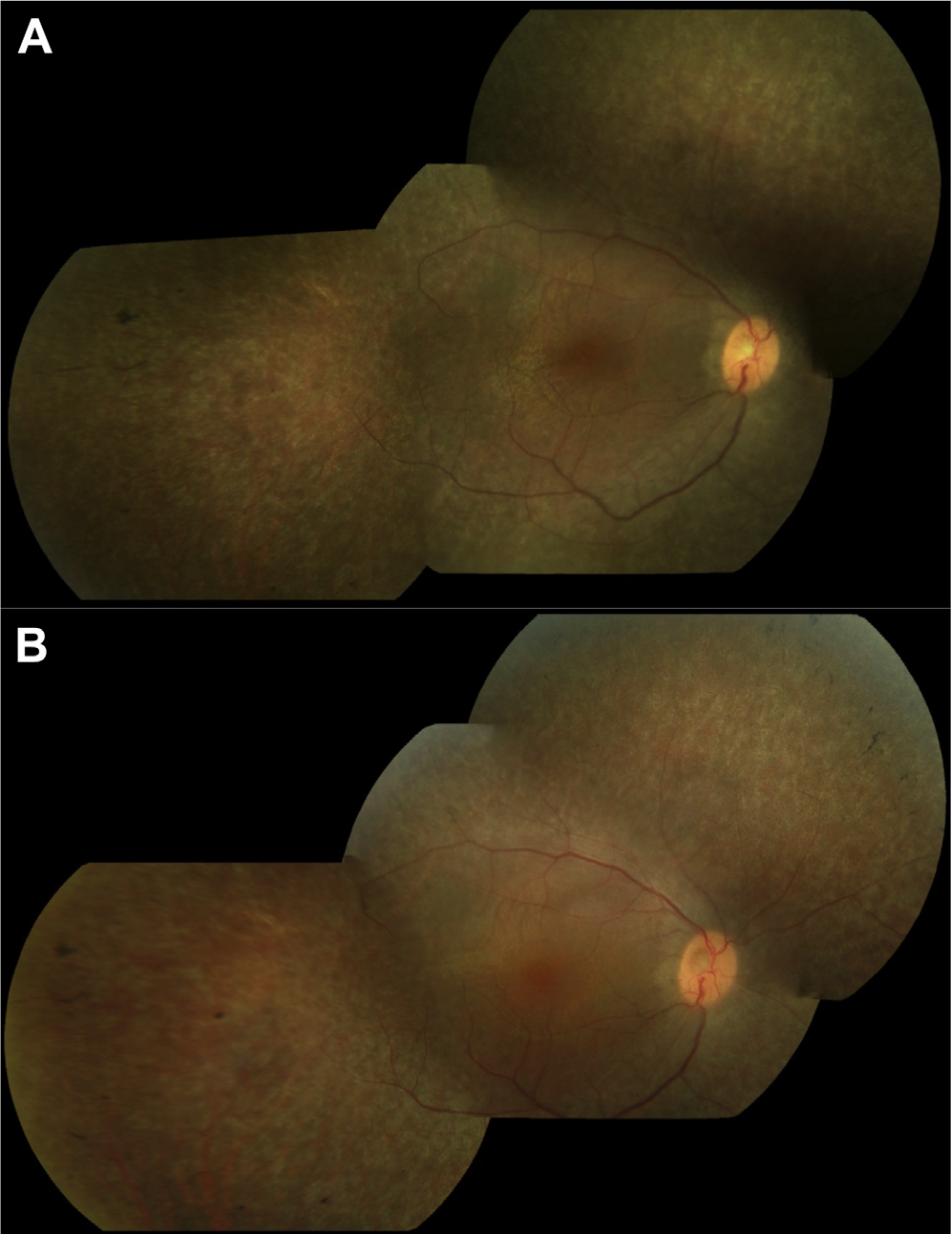
Color fundus photograph showing mild pigmentary changes in the right retina in patient 5c at 23 years of age **(A)** and increased pigmentary change 4 years later **(B)**.

**Figure 4:**
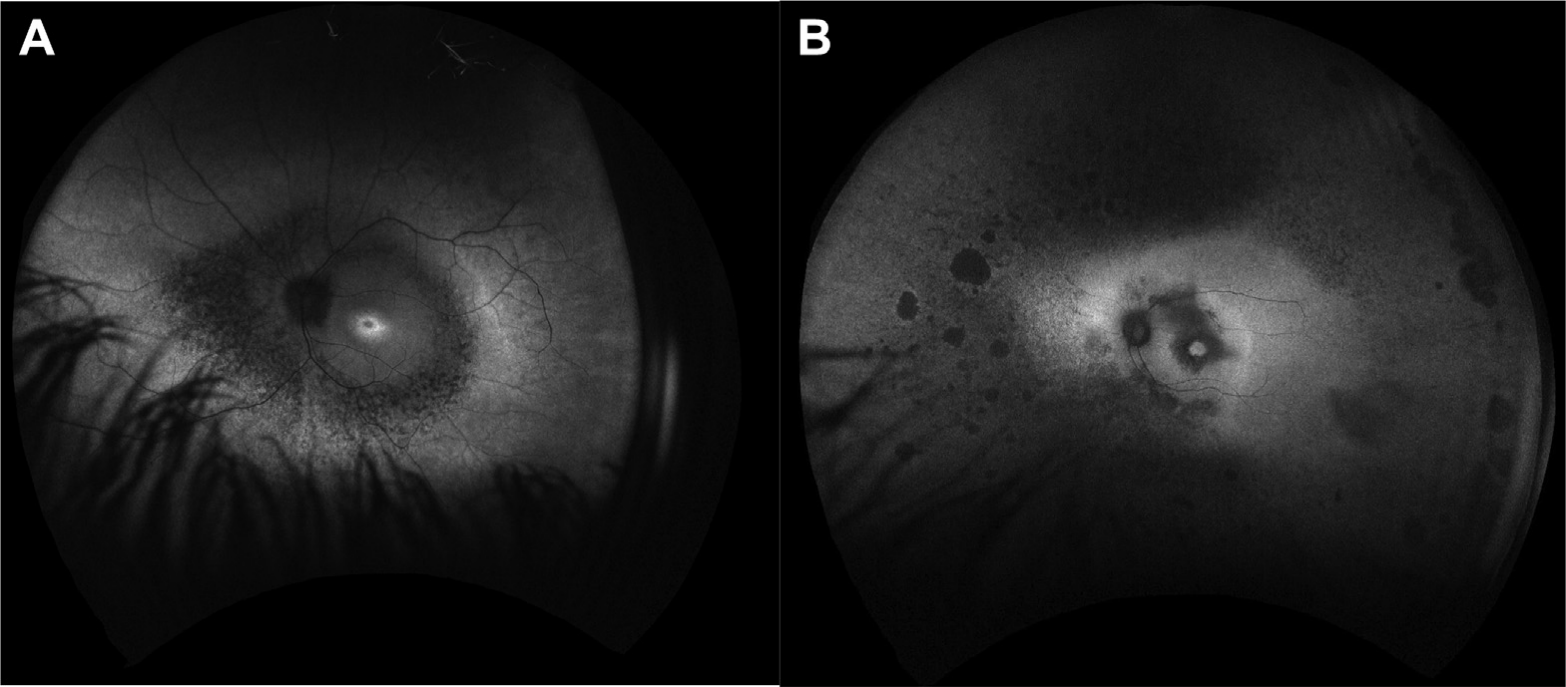
Optos fundus autofluorescence (FAF) of the left eye of patient 25 **(A)** showing central hyperautofluorescent ring with peripheral loss of autofluorescence compared with Optos FAF of patient 22a **(B)** showing loss of autofluorescence in the far periphery and in the paramacula region.

**Figure 5:**
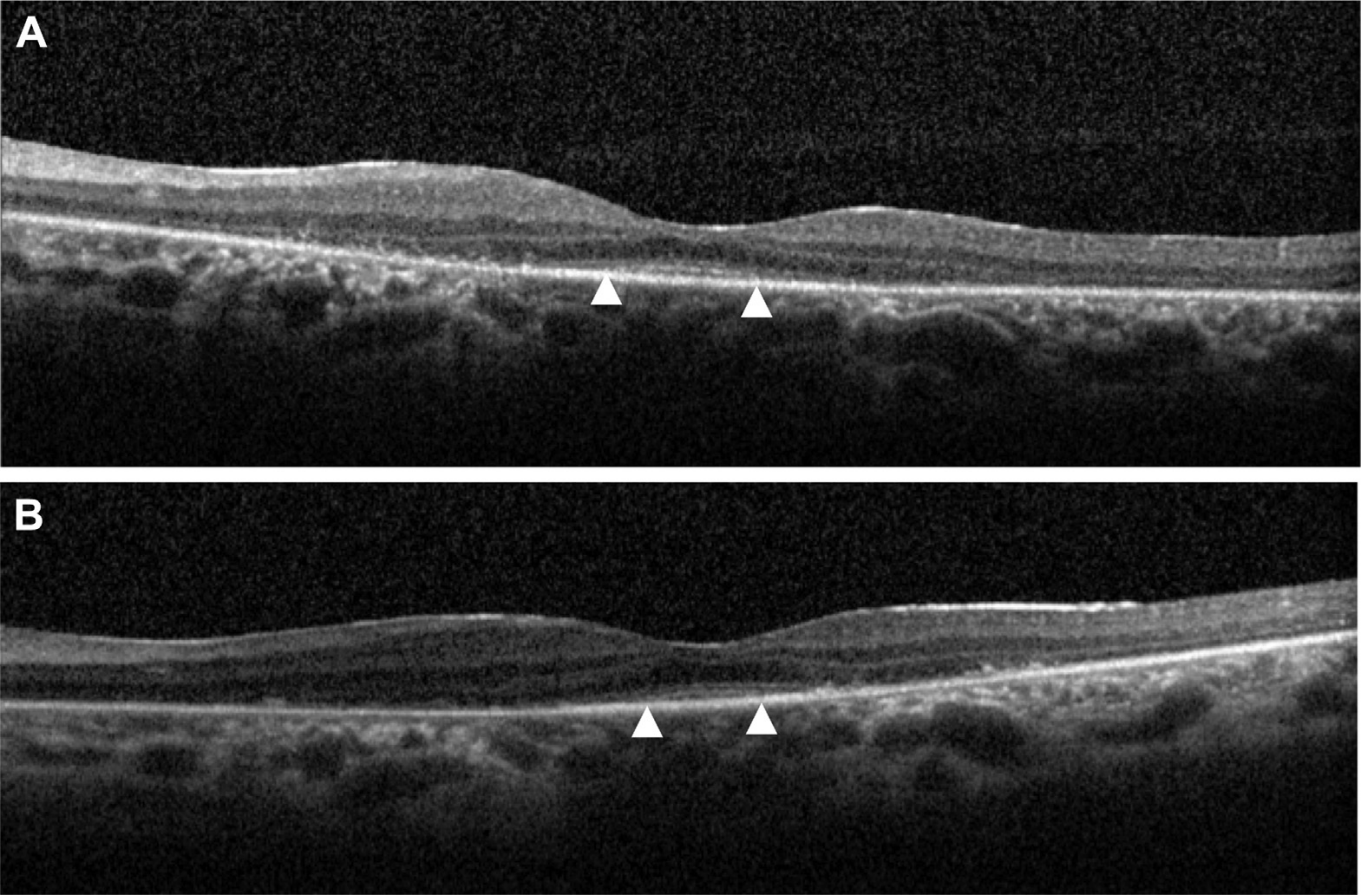
The OCT images of patient 22a showing preservation of left foveal outer retinal architecture at 36 years of age **(A)** and progression over 6 years **(B)**. *Triangles* denote termination of inner/outer segment line.

**Figure 6:**
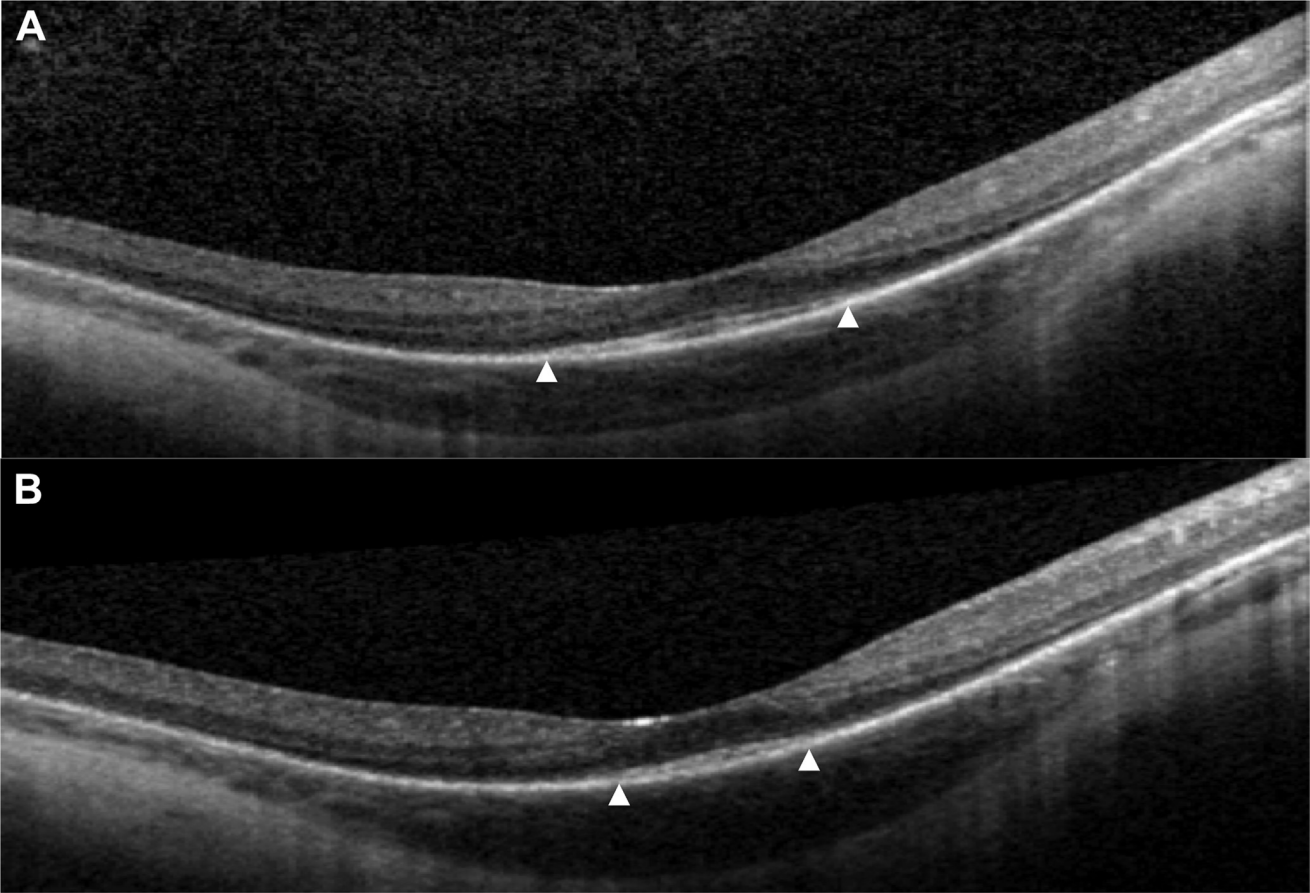
The OCT images of the right eye of patient 25 at 14 **(A)** and 18 years of age **(B)** showing progressive loss of the inner segment/outer segment line (*triangle*).

**Figure 7:**
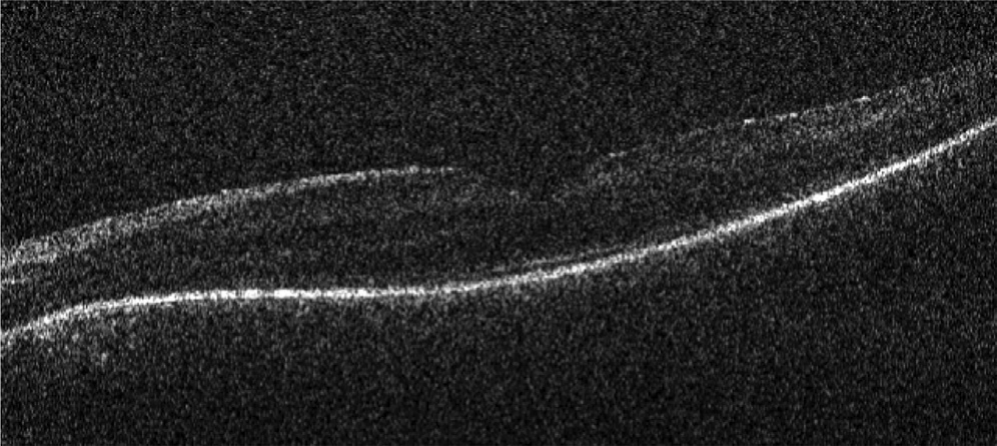
Handheld Bioptigen (Morrisville, NC) OCT imaging of the left eye of patient 33 (22 months old) that revealed relatively intact outer retinal structure.

Findings are summarized in Table 3, Table 4, Table 5, Table 6 and Figure 2, Figure 3, Figure 4, Figure 5, Figure 6, Figure 7. All patients had reduced vision or nystagmus noted within the first 4 years of life. Visual acuity loss was marked and of early onset, with 18% (n = 7) of patients having no perception of light in both eyes at presentation. Forty percent (n = 16) of all patients were able to record a VA on a Snellen chart, with 7.5% (n = 3) having a VA of better or equal to 6/15 in their better seeing eye and 80% (n = 32) having a VA of worse or equal to 6/60. In the 12 patients with serial VA measurement (Table 5), 4 (33%) had worsening of their VA over time. Two patients (17%) had visual deterioration in both eyes, 1 deteriorated from 6/18 right and 6/12 left to 6/36 in both eyes between 35 and 50 years of age, and 1 deteriorated from 6/30 to 2/60 in both eyes between 3 and 7 years of age. Two patients had deterioration in vision in the worst seeing eye only, 1 from 6/36 to 6/150 between 25 and 42 years of age, whereas the best-seeing eye was maintained at 6/30, and the other went from perception of light in both eyes to no perception of light in 1 eye. There were no patients with light perception or better VA at presentation, who subsequently progressed to no light perception in both eyes during follow-up assessment. Patients harboring 1 or more missense mutations or in-frame deletions had a final VA of 1/60 or better compared with those with 2 nonsense mutations who had a worse VA (75% vs. 26%) (Table 6).

**Table 3:** Clinical Findings in the *CEP290* Cohort

Patient (n)	40	
Age at presentation (median, range)	0	0–4
Female (n, percentage)	15	38%
Refraction (n, percentage)		
Hyperopia	19	48%
Myopia	1	3%
Plano	5	13%
Not available	15	38%
VA in best-seeing eye (n, percentage)		
No perception of light	7	18%
Perception of light	11	28%
Does not fix and follow	3	8%
Hand movements	1	3%
Fixate on large objects	2	5%
6/60–1/60	8	20%
6/48	1	3%
6/36	4	10%
6/15	1	3%
6/12	1	3%
6/9	1	3%
ERG (n, percentage)		
Extinguished	22	55%
Residual 30 Hz flicker	1	2.5%
Not available	17	42.5%
Systemic involvement (n, percentage)		
Joubert syndrome	1	3%
Renal failure	1	3%
Developmental delay/autism	6	15%
Other neurologic disorders	2	5%
Total	10	25%

ERG = electroretinogram; VA = visual acuity.

**Table 4:** Retinal Findings and Association with Age of Examination

Retinal Findings	n	Percentage	Mean Age, yrs	SD
Pigmentary retinopathy	16	40%	19.7	14.6
White flecks only	7	18%	5.9	4.3
Normal	17	43%	1.9	4.8

SD = standard deviation.

**Table 5:** Serial Measurement of Visual Acuity in Those with Longitudinal Records

Patient No.	Initial VA		Age at Initial Examination	Final VA		Age at Final Examination	Length of Follow-up (yrs)
	Right Eye	Left Eye		Right Eye	Left Eye		
3	PL	PL	29	NPL	PL	33	4
4	6/36	6/36	7	6/150	6/30	42	35
5a	6/60	6/60	12	6/48	6/60	33	21
5c	6/60	6/60	10	6/60	6/60	34	24
5d	6/60	6/60	19	6/60	6/60	29	10
6	NPL	NPL	5	NPL	NPL	11	6
10	6/30	6/30	3	2/60	2/60	7	4
12	PL	PL	0	PL	PL	15	15
17	NPL	NPL	2	NPL	NPL	5	3
22a	6/18	6/12	35	6/36	6/36	50	15
29	PL	PL	0	PL	PL	4	4
30	6/30	6/30	4	6/12	6/12	15	11

NPL = no light perception; PL = light perception; VA = visual acuity.

**Table 6:** Association between Predicted Amino Acid Effect and Final Visual Acuity, Excluding Those with Unknown Second Mutation

Mutations	Final VA	
	≥1/60	<1/60
≥1 missense mutations or an in-frame deletion	9	3
Both nonsense mutations	6	17

VA = visual acuity.

Of the 25 patients in whom refraction data were available, 19 (76%) were hyperopic, and only 1 (4%) was myopic. The remaining 5 patients did not have a significant refractive error.

Typical fundus images are shown in Figures 2 and 3. A normal fundus examination was observed in 43% (n = 17) of patients, 17% (n = 7) had white flecks in the periphery, and peripheral pigmentary changes were seen in 40% (n = 16) (Table 4). On the basis of the last recorded fundus examination findings, those with normal fundi (mean, 1.9 years; range, 0–21 years; standard deviation [SD], 5 years) tended to be the youngest, followed by those with white flecks (mean, 5.9 years; range, 2–13 years; SD, 4.3 years) and those with pigmentary retinopathy (mean, 19.7 years; range, 1–54 years; SD, 14.6 years), suggesting the evolution of the retinal phenotype from normal or fleck retinopathy to pigmentary retinopathy over time. However, longitudinal data were not available to show the sequential changes in retinal phenotype over time in the same patient. Figure 3 shows the progressive increase in pigment in the peripheral retina over 4 years in an 18-year-old patient.

A nonocular condition was present in 25% of patients. The most common association was a delay in development or autism (15%). Joubert syndrome and renal disease were uncommon, with only 1 patient (3%) with each condition in our cohort. This may represent an ascertainment bias because patients were recruited from a stand-alone eye hospital. All patients with nonocular condition have 2 nonsense *CEP290* mutations.

### Retinal Imaging

Thirteen patients (31%) had OCT and FAF imaging with the Spectralis imaging system. On FAF imaging, 10 of 12 patients (83%) had a perifoveal hyperautofluorescent ring, with peripheral loss of FAF (Fig 4). The remaining 2 patients (17%) had parafoveal and peripheral loss of FAF, with preservation of FAF at the fovea (Fig 5).

Eleven of 12 patients (92%) had relative preservation of foveal architecture, with a loss of peripheral macular outer retinal structure on OCT imaging (Figs 5 and 6). The remaining subject had total loss of the outer retina. Eight patients had serial OCT imaging, with 2 patients showing evidence of progressive loss of the inner segment ellipsoid line over time (Figs 5 and 6). The youngest patient in the series, patient 33 (22 months old) had handheld Bioptigen (Morrisville, NC) OCT imaging that revealed relatively intact outer retinal structure (Fig 7).

### Electrophysiologic Assessment

All young patients tested with skin electrodes and 2 adult patients (27 and 30 years of age) tested with corneal electrodes had undetectable photopic and scotopic ERGs in keeping with a severe photoreceptor dystrophy. One other adult patient had undetectable photopic (light adaptation 3.0 and 30 Hz) ERGs and grossly abnormal scotopic (dark adaptation 0.01 and 10.0) ERGs at the age of 34 years that worsened over the following 11 years, consistent with a progressive cone-rod dystrophy. In 1 adult, the PERG was technically poor because of nystagmus, but in 2 others who underwent testing (1 adult and 1 child aged 20 months), the PERG was undetectable in keeping with severe macular involvement.

## Discussion

Mutations in *CEP290* account for 15% to 20% of all cases of LCA/EOSRD.^9^ In this study, we describe a large group of patients who have undergone detailed clinical phenotyping at a single institution. The majority of patients in this cohort had severe visual loss at baseline, and in those with longitudinal data, many did not show deterioration in their VA. Hyperopia was the most common refractive error, and this is consistent with other forms of LCA.^25^ The retinal appearance was variable, with younger subjects being more likely to have a normal fundus appearance or peripheral white flecks without pigmentation. Older patients commonly showed evidence of peripheral retinal pigment migration. This variation with age has been reported in other studies^7^, ^9^, ^10^, ^26^ and suggests that, in contrast to the relatively stable central retinal function, there may be progressive peripheral photoreceptor cell death.

Despite a profoundly abnormal or extinguished ERG indicating severe global impairment of outer retinal function, we found that the outer retinal structure (inner segment ellipsoid and outer nuclear layers) at the fovea on OCT appeared to be relatively well preserved. This is consistent with findings in other studies.^7^, ^27^, ^28^ In particular, a recent study reported the presence of central photoreceptors in a cohort of patients with *CEP290* LCA, similar to our findings.^28^ The same study also showed a loss of rod function in these patients, which agrees with our ERG findings.^28^ This leads to optimism that restoration of cone function may be possible using gene therapy-based approaches.^29^ Such potential therapeutic approaches have been explored in vitro with a lentiviral vector containing human *CEP290* and has been shown to effectively transduce patient-specific induced pluripotent stem cell-derived photoreceptor precursor cells and rescue the cellular phenotype.^17^ Other studies have focused on the common deep intronic *CEP290* mutation, which creates a viable cryptic splice donor site that leads to the insertion of an additional exon that includes a sequence that encodes for a premature stop codon. These approaches have included the use of antisense oligonucleotide-mediated exon skipping to abrogate the disease-causing variant or correction of the splice defect using CRISPR/Cas9-mediated gene editing.^15^, ^18^, ^19^ Both of these novel approaches show great promise and human clinical trials are anticipated in the near future.

There are several potential challenges in the design of therapeutic trials for *CEP290* LCA, including eligibility criteria and outcome measures. Visual acuity will likely be insensitive partly because of the often poor VA at baseline. An improvement in ocular motor control and in the ability to navigate a standardized and validated mobility course may be useful functional measures. A global measure of light sensitivity, such as the full-field light sensitivity threshold test, which has been developed for patients with very poor vision, also may be valuable. Pupillometry may be informative, whereas electrophysiologic assessment is unlikely to be adequately sensitive, with additional reliability/feasibility challenges due to nystagmus. Spectral domain OCT will be valuable in determining any changes in foveal structure, although image acquisition may be challenging given poor fixation/nystagmus.

In patients with the milder form of LCA/EOSRD, caused by mutations in *RPE65*, gene therapy results in an improvement in visual function, potentially to a greater extent in younger patients.^30^ However, LCA-*CEP290* is associated with far more profound visual impairment at an earlier age (from birth/early infancy) compared with *RPE65* deficiency; therefore, visual cortical plasticity may be a more significant limitation to treatment response. Novel therapies may need to be given in infancy in most subjects to achieve the best visual outcome.

The majority of patients in our cohort had ocular involvement only. Joubert syndrome was diagnosed in 1 patient, and 1 patient had renal failure. Neurologic disorders (seizures and microcephaly), developmental delay, and autism were the most common nonocular features found in this cohort. The fact that the subjects were recruited from an eye hospital, and visual loss was the predominant early symptom, may have led to some ascertainment basis.

The deep intronic mutation, c.2991+1655A>G, was the most common variant in our cohort, with 55% of patients harboring it in the heterozygous or homozygous state, which is consistent with previous reports.^7^ Of note, missense variants were rare. A review of all *CEP290* mutations reported as “pathogenic” or “likely pathogenic” on the ClinVar database showed that only 2 of 89 such mutations were missense mutations.^31^ There were no missense variants in another large cohort.^7^ This suggests that missense variants are well tolerated and do not sufficiently abrogate CEP290 protein structure or function, or that the reported missense variants in LCA-*CEP290* may functionally act like null alleles. A study on the effect of *CEP290* in vitro showed that full-length CEP290 protein exhibited attenuated activity when compared with truncation mutants lacking the N or C terminus, suggesting the N and C terminus of the CEP290 protein have a regulatory effect to explain the pathogenic effect of nonsense mutations.^32^ Consistent with this, in our study, patients with 2 nonsense *CEP290* mutations had a worse final VA. Furthermore, all patients with systemic features had 2 nonsense *CEP290* mutations in our cohort.

Early molecular diagnosis of *CEP290-*related retinal dystrophy is critically important, because this allows the provision of better informed advice on prognosis and will prompt further investigation to rule out associated systemic disease. Furthermore, novel therapies for LCA-*CEP290* are under development and clinical trials are anticipated in the near future. Our study provides key information about the clinical phenotype and natural history of LCA-*CEP290,* which will help inform patient selection and study design for such trials.
